# Biomass Waste Utilization as Nanocomposite Anodes through Conductive Polymers Strengthened SiO_2_/C from *Streblus asper* Leaves for Sustainable Energy Storages

**DOI:** 10.3390/polym16101414

**Published:** 2024-05-16

**Authors:** Thanapat Autthawong, Natthakan Ratsameetammajak, Kittiched Khunpakdee, Mitsutaka Haruta, Torranin Chairuangsri, Thapanee Sarakonsri

**Affiliations:** 1 Office of Research Administration, Chiang Mai University, Muang, Chiang Mai 50200, Thailand; thanapat.a@cmu.ac.th; 2 Department of Chemistry, Faculty of Science, Chiang Mai University, Muang, Chiang Mai 50200, Thailand; natthakan_r@cmu.ac.th (N.R.); kittiched_kh@cmu.ac.th (K.K.); 3 Material Science Research Center, Faculty of Science, Chiang Mai University, Muang, Chiang Mai 50200, Thailand; 4 Center of Excellent for Innovation in Chemistry (PERCH-CIC), Faculty of Science, Chiang Mai University, Muang, Chiang Mai 50200, Thailand; 5 Institute for Chemical Research, Kyoto University, Kyoto 611-0011, Japan; haruta@eels.kuicr.kyoto-u.ac.jp; 6 Department of Industrial Chemistry, Faculty of Science, Chiang Mai University, Muang, Chiang Mai 50200, Thailand; torranin.c@cmu.ac.th

**Keywords:** waste recycling, biomass, silica–carbon, conductive polymer, nanocomposite, anode material, sustainable energy, lithium-ion battery

## Abstract

Sustainable anode materials, including natural silica and biomass-derived carbon materials, are gaining increasing attention in emerging energy storage applications. In this research, we highlighted a silica/carbon (SiO_2_/C) derived from *Streblus asper* leaf wastes using a simple method. Dried *Streblus asper* leaves, which have plenty of biomass in Thailand, have a unique leaf texture due to their high SiO_2_ content. We can convert these worthless leaves into SiO_2_/C nanocomposites in one step, producing eco-materials with distinctive microstructures that influence electrochemical energy storage performance. Through nanostructured design, SiO_2_/C is thoroughly covered by a well-connected framework of conductive hybrid polymers based on the sodium alginate–polypyrrole (SA-PPy) network, exhibiting impressive morphology and performance. In addition, an excellent electrically conductive SA-PPy network binds to the SiO_2_/C particle surface through crosslinker bonding, creating a flexible porous space that effectively facilitates the SiO_2_ large volume expansion. At a current density of 0.3 C, this synthesized SA-PPy@Nano-SiO_2_/C anode provides a high specific capacity of 756 mAh g^−1^ over 350 cycles, accounting for 99.7% of the theoretical specific capacity. At the high current of 1 C (758 mA g^−1^), a superior sustained cycle life of over 500 cycles was evidenced, with over 93% capacity retention. The research also highlighted the potential for this approach to be scaled up for commercial production, which could have a significant impact on the sustainability of the lithium-ion battery industry. Overall, the development of green nanocomposites along with polymers having a distinctive structure is an exciting area of research that has the potential to address some of the key challenges associated with lithium-ion batteries, such as capacity degradation and safety concerns, while also promoting sustainability and reducing environmental impact.

## 1. Introduction

Rechargeable lithium-ion batteries (LIBs) are widely used as state-of-the-art energy storage systems, especially in portable electronic devices, renewable energy storage systems, and electric vehicles [1,2]. Higher energy and power density in LIBs are now being demanded by the marketplace, which is mainly dictated by the materials employed. Graphite is the most commonly used anode material in commercial LIBs; however, it has a relatively low specific capacity of 372 mAh g^−1^, which cannot meet the demands of high-energy LIBs [3]. Even though graphite has a high electrode/electrolyte interphase, is low-cost, and has an acceptable cycle life, its low operating voltage can cause lithium dendrites to grow, which can cause a cell short circuit [4,5]. Because of the limitations of graphite anodes, this has led to the exploration of innovative anode materials, such as silicon, tin, and lithium metal, which have higher theoretical capacities and the potential for higher energy densities. Nevertheless, these materials also have their own challenges, such as volume expansion, dendrite formation, capacity fading, and safety concerns, which need to be addressed to enable their commercialization. With high theoretical capacities and improved electrochemical performance expected to be achieved to satisfy the needs of LIBs, overall, developing innovative anode materials is an ongoing research focus in the field of LIBs.

Among several anode materials explored, silicon (Si) has the highest theoretical capacity for the Li_4.4_Si phase (4200 mAh g^−1^). It is also abundant, safer, and has a substantially better operating potential (0.3 V vs. Li^+^/Li) than graphite [6]. Moreover, lithiated silicon is more stable than graphite in ordinary electrolytes. Therefore, it is a desirable anode candidate for LIBs. In the last few decades, silica (SiO_2_) has become an increasingly common alternate source for extracted Si anodes. This is because it has interesting anode properties, such as a relatively high lithium-ion storage capacity (1950 mAh g^−1^), low cost, and natural abundance. However, the realistic capacities of Si and SiO_2_ remain lower than their theoretical capacities because of the pulverization during the alloying and dealloying processes, resulting in electrode fracture and electrical contact loss, causing impedance increases and rapid capacity fading [7]. Furthermore, SiO_2_ has weaknesses such as poor conductivity and low Coulombic efficiency during the initial cycle that really should be addressed [8]. Many methods have been explored to overcome these issues, one of which is to prepare unique SiO_2_ nanostructures [9,10], and another is to develop SiO_2_-based composites [11,12]. Impressively, SiO_2_ nanocomposites with carbon gained better attention in recent anode investigations. In consequence, anodes suffer physical deterioration, which causes electrode cracking. Even when they are fabricated into composites, such as carbon, the mitigation of the issue remains insufficient. To achieve this critical challenge and limit, the anode material’s self-healing characteristics, which include conductive polymer coatings, will ideally repair cracks or regenerate their structure when damaged and prevent the formation of dendrites, causing short circuits and safety issues, thereby increasing their durability and extending their lifespan [13]. Reversible dynamic interactions in self-healing materials, such as hydrogen bonds [14,15], dynamic metal–ligand interaction [16], hydrophobic interaction [17], and dynamic covalent boroxine bonds [17], have recently provided numerous benefits for achieving robust mechanical properties and high-efficiency self-healing. Despite interactions, hydrogen bonds have been successfully implemented in LIBs because they have demonstrated great promise, are simple to design, fabricate, and functionalize, and have fewer requirements [13]. Intriguingly, self-healing materials based on hydrogen bonds have been effectively applied in LIBs, including supramolecular rubber (SR), ureido pyrimidinone (UPy)-based polymers, carboxylated polyurethane (CPU), polyacrylic acid (PAA), polyaniline (PANI), polypyrrole (PPy), polyacrylic acid (PAA), polythiophene (PT), sodium alginate (SA), polyacrylic acid (PAA), carboxymethylcellulose (CMC), and their derivatives [18,19,20,21]. Interestingly, the unique polymer matrix of PPy can reduce internal stress in solid particle anodes that undergo substantial volume changes during charge–discharge, thereby improving conductivity and significantly improving cycling performance compared to individual solid particles [22,23,24]. These composites have demonstrated PPy’s behavior as a surface coating layer and/or active matrix, enhancing structural stability and cycle performance in batteries. The PPy polymer improves the conductivity of the electrode by lowering the resistance between particles and stopping unwanted reactions between the active materials and electrolytes [25].

Recent research has paid the most attention to these materials, which have been introduced into SiO_2_-based anode materials derived from biomass [26,27]. SiO_2_ incorporation into conductive self-healing polymers improved their thermal stability, specific capacity, electrical contact, and cycle life [28]. These could be because of their high degree of flexibility, conductive backbone, and controllable electrical conductivity, which enable them to be cycled multiple times with little degradation [29]. Therefore, the development of nanocomposite anode materials [30] with self-healing features has the potential to address the key challenges associated with LIBs. However, more research is needed to optimize and ensure scalability and commercial viability.

A large number of studies are being done on different plants [31] and their waste biomass products [32] in order to develop natural carbon-based sources with numerous applications. *Streblus asper* Lour. (SAL), which grows abundantly in tropical regions, especially in Thailand, is an attractive plant because of its distinctive leaf texture. This plant is commonly known as “Koi”, “Siamese rough bush tree”, or “Toothbrush tree” in Thai [33]. During the dry season, this tree frequently sheds its leaves, resulting in a large amount of waste. As a result, most people would prefer to burn them, leading to air pollution and PM2.5 problems. Fortunately, these leaves have been considered sustainable and have potential as low-cost raw materials for the production of value-added materials. The rough texture of SAL leaves is caused by the high content of SiO_2_ [34]. In addition, the important supporting and transport organs mainly consist of high-carbon components, notably lignin, cellulose, and hemicellulose, which can be a source of carbon [31]. These natural biomass-derived carbon materials can preserve their unique microstructure, which influences electrochemical energy storage performance [35]. From the benefits of natural SiO_2_ and carbon, the SAL leaves can be effectively converted into SiO_2_/C composites as eco-materials in one step.

There are several challenges associated with anode materials in LIBs, including capacity degradation, safety concerns, cost, and environmental impact. Importantly, developing more sustainable and environmentally friendly anode materials is a critical challenge for industry. In this work, the use of green nanofillers (SiO_2_/C) derived from SAL leaves and conducting polymers (PPy), which were integrated into green polymeric nanocomposites, has been illuminated, whereas the SA brown algae were used as a natural binder. The SA-PPy integrates to have a robust framework and efficient self-healing behavior. Therefore, we have proposed a green nanocomposite design that is simple, cost-effective, and environmentally friendly to prepare PPy@Nano-SiO_2_/C nanocomposite from renewable resource materials through green strategies as anode materials for LIBs in two steps: The self-assembled SiO_2_ that uniformly exists within carbon was first derived from SAL leaves via thermal treatment. The PPy@Nano-SiO_2_/C nanocomposites were then produced through in situ polymerization of pyrrole monomers using iron (III) chloride as an initiator, affording the SiO_2_ nanoparticles in these synthesized composites dual protection and self-healing properties. Herein, we introduce the first report that utilizes *Streblus asper* leaves as a raw resource. The proportional effects and electrochemical properties of nanocomposites were examined. The derived natural carbon could be a great host for SiO_2_ nanoparticles, whereas SA-PPy can act as a stable and conductive network with better electrical conductivity and cycle stability. All these factors make SA-PPy@Nano-SiO_2_/C a promising green polymeric anode for cost-effective and sustainable LIBs.

## 2. Experimental Section

### 2.1. PPy@SiO_2_/C Nanocomposite Synthesis

The green nanocomposite fabrication of mechanically robust PPy@nano-SiO_2_/C anode materials utilizing waste recycling of SAL consists of three steps: (1) formation of SiO_2_ nanoparticles (Nano-SiO_2_) by calcination in the ambient; (2) co-assembly of SiO_2_ nanoparticles covered carbon (Nano-SiO_2_/C) by thermal treatment under inert gas; and (3) nano-compositing of SiO_2_/C and PPy by in situ polymerization to obtain PPy coated on Nano-SiO_2_/C. SAL leaves as biowastes were collected in the dry season at Chiang Mai University, Thailand. The fallen leaves were cleaned up and then sun-dried for two days as raw materials before being blended into dried powder. The rough size of the dry leaf powder after blending was first treated with 1 M HCl (37% HCl, RCI-Labscan, Bangkok, Thailand) to remove the metal impurities and digest the hemicellulose. The treated powder was then washed with deionized (DI) water to remove the excess HCl until the pH was neutral and dried in an oven. The treated powder was heated up to 800 °C for 2 h with a heating rate of 5 °C/min in a quartz tube furnace under an ambient atmosphere to provide SiO_2_ nanoparticles, denoted as “Nano-SiO_2_”. For comparison, the treated powder was also prepared via heat treatment under an inert N_2_ atmosphere to produce SiO_2_/C nanocomposite, denoted as “Nano-SiO_2_/C”. In this step, 10 g of dried leaves produced products with yields of approximately 53% and 20% after heat treatment with and without an inert atmosphere, respectively. From previous work, the use of heat treatment at 800 °C is the most optimum condition. The hydrocarbon compound in the form of hemicellulose in the SAL would completely be oxidized in the air, releasing the CO_2_ to remain only SiO_2_ in this state. In contrast, the hydrocarbon compound would not decompose into CO_2_ when heated under an inert atmosphere; instead, at this temperature, it would yield carbon, which, in this state, can produce SiO_2_/C in a single step. After that, the fine SiO_2_/C powder was homogeneously dispersed in the 95% EtOH (C_2_H_6_O, QrëC, Bangkok, Thailand). Following that, pyrrole monomer (Py) (98% C_4_H_4_NH, Sigma-Aldrich Co., Ltd., Burlington, MA, USA) was added to the homogeneous suspension in a weight ratio of 1:0.5 (SiO_2_/C:Py). This mixed suspension was continuously stirred, followed by sonication at room temperature, to obtain a homogeneous suspension. It was then added dropwise with ten times the amount of ferric trichloride hexahydrate (FeCl_3_·6H_2_O, KEMAUS, Sydney, Australia) as an initiator to promote in situ polymerization. This polymerized suspension was continually stirred for 12 h at room temperature. The polymerized product was collected and washed with DI water to eliminate any excess FeCl_3_·6H_2_O. The polymerized product was dried at 60 °C overnight. Finally, the final product of SiO_2_/C nanocomposited with polypyrrole (PPy) is referred to as “PPy@Nano-SiO_2_/C”.

### 2.2. Materials Characterization

The powder X-ray diffraction (XRD) patterns were performed using a Rigaku Miniflex II desktop (Tokyo, Japan) diffractometer using Cu Kα radiation to analyze the structure and crystallinity. A Fourier transform infrared spectrometer (FT-IR, Tensor 27, Bruker, Billerica, MA, USA) was used to record the spectra of prepared materials using attenuated total reflectance (ATR) mode. Raman spectra were recorded on a Jobin Yvon Horiba, model T64000, for the analysis of the structural fingerprints in the as-prepared product. Thermogravimetric analysis (TGA, Rigaku, Thermo Plus EVO2, Tokyo, Japan) was conducted on a Mettler Toledo TGA/DSC 3+ HT1600 with a heating rate of 10 °C/min from ambient temperature to 1000 °C in air to determine the carbon content. The microstructure, morphology, and crystalline phase of the preparative nanocomposites were examined by using scanning electron microscopy (SEM, JEOL JSM-7800F-Prime, Tokyo, Japan) with secondary electron and backscattering detectors and transmission electron microscopy (TEM, JEOL JEM-2100 PLUS, Tokyo, Japan). High-resolution and scanning transmission electron microscopy equipped with energy dispersive spectroscopy (HRTEM and STEM-EDS, JEOL JEM-ARM200F, Tokyo, Japan) was employed to examine the lattice fringes and graphitization in the as-prepared product. To study the surface area and the pore size distribution, gas sorption isotherms of N_2_ were measured by Quantachrome Model Autosorb 1 MP (Giangarlo Scientific Co. Inc., Pittsburgh, PA, USA) using the Brunauer–Emmett–Teller method and Barrett–Joyner–Halenda (BJH) theory. In order to ascertain the durability of the electrolyte used in the experiment, a UV-visible spectrophotometer (Thermo Scientific™–Evolution 201, Middleton, WI, USA) was employed.

### 2.3. Electrochemical Measurements

The working electrode was produced by combining prepared powders as an active material, sodium alginate (SA, Loba chemie Pvt. Ltd., Mumbai, India) as a binder, and Super-P (Xiamen TOB New Energy Technology Co., Ltd., Xiamen, Fujian, China) as a conductive agent in an aqueous solvent in a weight ratio of 60:20:20. The combined slurry was evenly plastered over a copper foil current collector using a doctor blade and then dried under vacuum at 80 °C for 12 h to obtain an electrode, referred to as “SA-PPy@Nano-SiO_2_/C”. In a dry argon glove box, a coin cell (CR2016) was assembled, consisting of the working electrode and lithium metal as a reference electrode, whereas the Celgard 2400 is employed as the separator. The organic electrolyte was 1 M LiPF_6_ dissolved in ethylene carbonate/dimethyl carbonate (EC)/(DMC) (1:1 by vol.%) (Sigma Aldrich Co., Ltd., Burlington, MA, USA). The battery performance of the fabricated coin cells, including galvanostatic charge and discharge profiles, rate capability, and cycle stability, was evaluated at various current rates in the potential range of (0.01–3.0 V vs. Li/Li^+^) on a Neware (BTS-4000, Shenzhen, China) battery testing system at room temperature. The current densities were calculated based on the loading mass and theoretical specific capacity of the anode active material. Cyclic voltammetry (CV) and electrochemical impedance spectroscopy (EIS) were also recorded at constant room temperature utilizing potentiostat/galvanostat equipment (Autolab PGSTAT302N, Herisau, Switzerland).

## 3. Results and Discussion

### 3.1. Synthesized Materials Characterization

**Figure 1:**
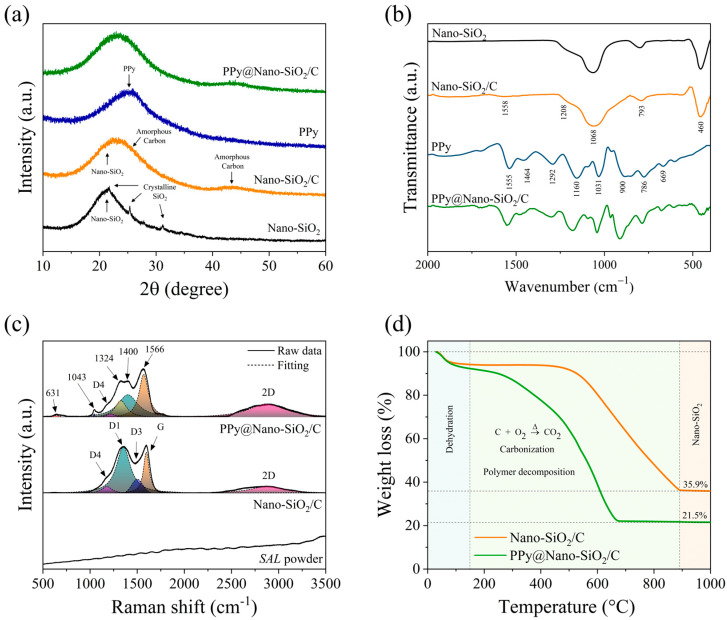
(**a**) XRD patterns and (**b**) FTIR spectra of synthesized products: Nano-SiO_2_, Nano-SiO_2_/C, PPy, and PPy@Nano-SiO_2_/C nanocomposites; (**c**) Raman spectra; and (**d**) thermogravimetric analysis curves under an air atmosphere for synthesized Nano-SiO_2_/C and PPy@Nano-SiO_2_/C nanocomposites.

The phase formation and crystallinity of synthesized products, including Nano-Silica, Nano-SiO_2_/C, PPy, and PPy@Nano-SiO_2_/C nanocomposites, were investigated using the XRD technique as depicted in Figure 1a. For the as-synthesized Nano-SiO_2_ sample, the XRD patterns were observed to have a broad peak near 23° and small overlapped crystalline patterns at 21.83°, 28.33°, and 31.29°. These patterns corresponded to the characteristic patterns of amorphous SiO_2_ combined with some low-crystalline SiO_2_ (α-cristobalite, JCPDS No. 39-1425) [36], respectively. After co-synthesis of SiO_2_ and carbon, there were only two broad peaks centered at 26° and 43°, corresponding to the carbon planes (JCPDS No. 41-1487) of (002) and (100), respectively. These indicated the presence of amorphous carbon or low crystalline carbon. Thus, the precise carbon structures in all synthesized samples would be investigated in greater depth by HR-TEM and SAED techniques. Evidently, the amorphous and crystalline SiO_2_ patterns disappeared completely. This could be due to the intensity-increasing overlapping of carbon patterns. On the other hand, the XRD patterns of pure PPy exhibited only a broad peak at 25° centered. This indicates that the nature of the as-synthesized PPy is amorphous. For the XRD patterns of PPy@Nano-SiO_2_/C, there were also only two broad peaks, which are mainly influenced by carbon. However, because of the nanocomposite formation, the two broad peaks could occur due to overlapping patterns of SiO_2_, carbon, and PPy. Compared to the two XRD patterns of Nano-SiO_2_/C and PPy@Nano-SiO_2_/C, there was no discernible peak displacement in the XRD patterns. More importantly, the center-highest intensity peak value of the prepared nanocomposite material (23.11°) is positioned between the two overlapped broadening peaks of Nano-SiO_2_/C and PPy, which are centered at 22.22° and 25.88°, respectively. This indicates that the structure of SiO_2_ and carbon was unaffected by the addition of PPy.

To evaluate the successful polymerization of pyrrole monomer and the existence of PPy following in situ polymerization, the characteristic patterns of PPy were examined by FTIR. Figure 1b illustrates the FTIR spectra of all synthesized materials. For the chemical bonding characterization, the FTIR spectra of as-synthesized materials were obtained in the appropriate range of 2000–400 cm^−1^. Recently, the characteristic FTIR peaks of PPy were reported by Sanches et al. [37]. Therefore, the FTIR spectrum of synthesized pure PPy was also given for reference and compared in this work. Both the 786 cm^−1^ and 900 cm^−1^ bands were attributed to C–H stretching. Intriguingly, the low-intensity peak at 786 cm^−1^ revealed a predominance of α-carbon atoms in the PPy ring, coinciding with the ordered planar configuration of PPy, whereas Py rings are linked sequentially via α–α connections, whereas alternative connections can lead to disorder in the polymer chain [38]. This phenomenon is characteristic of conjugated polymers, whose electrical conductivity is primarily influenced by carrier concentration and mobility. A high α–α connection ratio signifies a high degree of order, which facilitates carrier migration [39]. A band at 1031 cm^−1^ can be attributed to the =C–H vibration in-plane deformation. C–H in-plane and out-of-plane deformations are responsible for the band at 1160 cm^−1^. Bands at 1464 cm^−1^ and 1555 cm^−1^ were attributed, respectively, to the vibration of the pyrrole ring (C–N) and the ring stretching mode (C=C, C–C). The bands at 1206 cm^−1^ and 1292 cm^−1^ were designated as the N–C stretching band and =C–H in-plane vibration, respectively. Focusing on PPy@Nano-SiO_2_/C, the spectra are unambiguous proof that the combination of Nano-SiO_2_/C and the PPy spectra pattern actually occurred. Therefore, the bands that were detected in the PPy@Nano-SiO_2_/C nanocomposite of this investigation recognize the polymerization of pyrrole in the nanocomposite and are compatible with those previously reported in the scientific literature [37,40].

As shown in Figure 1c, room-temperature Raman spectroscopy of the produced materials, including SAL powder, Nano-SiO_2_/C, and PPy@Nano-SiO_2_/C, revealed diverse bands between 500 and 3500 cm^−1^. The degree of graphitization and band vibrations were measured to identify structural changes from SAL powder, a raw material, to synthesized nanocomposites (Nano-SiO_2_/C and PPy@Nano-SiO_2_/C). The three major peaks—the D band, G band, and 2D band—in the Raman spectrum indicated the existence of graphitic carbon. According to the research of Eshun, J. et al. [41], the D band at 1350 cm^−1^ is due to defect structures in highly organized carbonaceous materials, which correspond not only to the condensed benzene rings in amorphous carbon, but are also associated with the vibrating modes of disordered graphite rings. In addition, the G band identified at roughly 1580 cm^−1^ provides evidence of the vibrations of sp^2^ carbon atoms, whereas double bonds are in graphitic materials. These are a result of the carbon aromatic ring systems present in pristine structures. In particular, Raman spectra determined that 2D bands detected at 2850 cm^−1^ are indicative of graphitic structures’ unique multilayer characteristics. Interestingly, the spectra of Nano-SiO_2_/C nanocomposite contained upwards of a single D band, which was consistent with the prior study [42]. The D band was modeled using Lorentzian equations that resulted in three peaks (D1, D3, and D4). In contrast to the most prominent D1 band at 1350 cm^−1^, the D3 band was identified at roughly 1500 cm^−1^ and the D4 band was discovered as a shoulder peak at approximately 1200 cm^−1^. It is hypothesized that the D3 band reflects sp^2^-bonded carbon fragments or functional groups within the disordered structure, whereas the D4 band is devoted to the vibration modes of sp^3^ carbon. Taking into account the SAL powder and Nano-SiO_2_/C, the obtained carbon in the SAL raw materials, which was not detected in Raman spectra, was transformed into sp^2^ and sp^3^ carbons in the graphitic structure, despite the disordered nature of the material. Due to the influence of PPy addition, the D band shape of PPy@Nano-SiO_2_ was altered and split into more than four peaks, which overlapped with the D band in Nano-SiO_2_/C. This outcome assigned to predominant band vibrations in PPy is consistent with what has previously been reported in the scientific literature [43]. C–C in-ring, antisymmetric C–N stretching, C–H bending, and N–H bending stretching were attributed to the peak at 1400 cm^−1^. The peak at 1324 cm^−1^ was assigned to antisymmetric C–N stretching and C–C in-ring stretching. The designation 1043 cm^−1^ was a C–H in-plane distortion. The peak at 631 cm^−1^ was finally classified as a C–C ring torsional. In accordance with the FTIR discovery, our analyses strongly corroborate the effective synthesis of PPy, whose molecular chains consist primarily of α–α links in Nano-SiO_2_/C to achieve the PPy@Nano-SiO_2_/C nanocomposite.

Employing TGA analysis, the weight content of as-prepared nanocomposites was investigated. TGA entails the measurement of a change in mass by subjecting samples to a predetermined temperature regimen. This measurement was conducted in an environment ranging from 25 to 1000 °C in an ambient atmosphere, as shown in Figure 1d. Generally, when the temperature was up to 150 °C, the remaining moisture would be totally evaporated. Afterward, the sample predominantly lost weight due to thermal and oxidative degradation. After that, the steady weight loss proceeded exclusively in PPy@Nano-SiO_2_/C at temperatures ranging from 280 °C to 800 °C, which can be attributed to PPy oxidation [44]. This suggested that the residual PPy had been degraded, decreasing the amount of carbon bonded to PPy polymer chains and removing more permanent nitrogen-doped units from the PPy ring, both of which are evident from the FTIR study. Unfortunately, carbon in all its forms is eliminated from oxidizing when the temperature rises to 900 °C. The significant weight loss observed at temperatures above 500 °C appears to be primarily attributable to the pyrolysis and carbonization of the carbon backbone chain from PPy and carbon, resulting in the release of CO_2_ [40]. Eventually, the TGA curves demonstrated that the prepared nanocomposites disintegrated completely, although the weight loss of nanocomposite products remained rather steady beyond 900 °C. This suggests that the leftover white product of nanocomposites after high-temperature examination was only the incomplete combustion product of SiO_2_, which corresponds to their superior thermal stability. For the Nano-SiO_2_ sample, it is presumed that this sample consisted primarily of SiO_2_. In addition, there is no silica loss throughout the entire production process because SiO_2_ is chemically inert and stable in the ambient environment. The SiO_2_ contents of Nano-SiO_2_/C and PPy@Nano-SiO_2_/C were determined to be 38.12 and 23.30 wt.%, whereas the carbon contents were 61.88 and 37.82 wt.%. Consequently, the PPy composition for PPy@Nano-SiO_2_/C was 38.88 wt.%. Therefore, using these TGA results, the weight loss data of as-prepared nanocomposites are summarized, and the theoretical specific capacity is calculated in Table 1.

**Figure 2:**
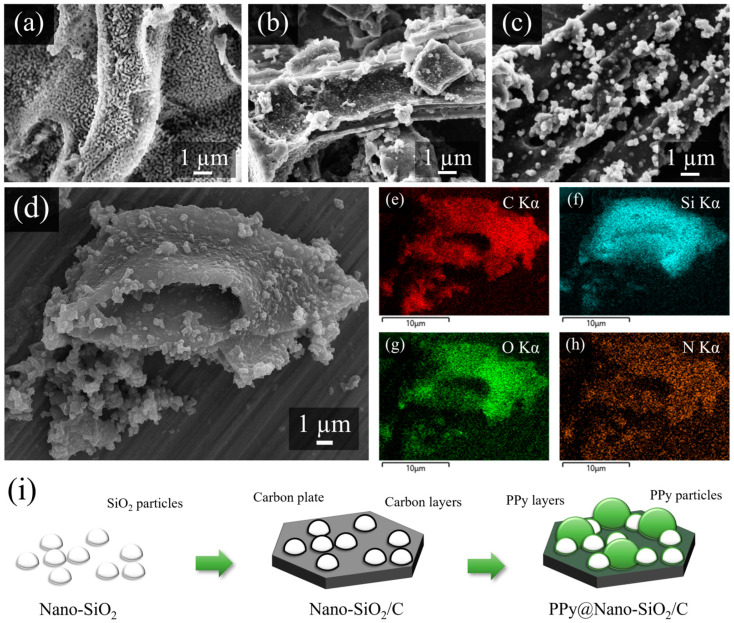
SEM images of the as-prepared products: (**a**) Nano-SiO_2_, (**b**) Nano-SiO_2_/C, and (**c**) PPy@Nano-SiO_2_/C nanocomposites; (**d**) the SEM-EDS mapping area corresponded to the elemental mapping of the PPy@Nano-SiO_2_/C nanocomposite: the corresponding elemental mapping of (**e**) C, (**f**) Si, (**g**) O, and (**h**) N, respectively; (**i**) schematic diagram of morphology in as-synthesized materials.

Employing SEM investigation, the morphology of synthesized products was analyzed; the results are presented in Figure 2a–c. Due to the natural structure of SAL, the morphology of Nano-SiO_2_ was observed to be a coral-like structure composed of small individual SiO_2_ nanoparticles, as illustrated in Figure 2a. As depicted in Figure 2b, the structure of Nano-SiO_2_/C contains carbon layers because of the carbonization of hemicellulose in the SAL. This suggests that the open space obtained from the coral-like Nano-SiO_2_ was filled and covered by carbon, which had a layered structure in the Nano-SiO_2_/C nanocomposite. Figure 2c demonstrates that, as a direct result, the morphology of the PPy@SiO_2_/C sample was altered. After incorporating PPy into Nano-SiO_2_/C, an SEM image of the PPy@SiO_2_/C nanocomposite revealed that the material possessed a thicker sheet adorned with well-distributed particles, which may well be PPy formation during the in situ polymerization process. Considering the thick layer observation, it can be concluded that the PPy is successfully encapsulated on the Nano-SiO_2_/C. This characteristic structure significantly improves the materials’ surface activity, and PPy greatly enhances the materials’ electrical conductivity. The PPy@SiO_2_/C nanocomposite evident in the image enables the nanocomposite to offer double protection against the volume expansion of SiO_2_.

This study addresses energy-dispersive X-ray spectroscopy mapping, where additional examination of element distribution in PPy@Nano-SiO_2_/C can be conducted. The EDS mapping acquired the element signals from the captured area of the SEM image, as shown in Figure 2d, demonstrating that the elements C, Si, O, and N, respectively (Figure 2e–h), were uniformly distributed throughout the material. The EDS mapping identifies the presence of N, and it is highly probable that the predominance of nitrogen (N) sources from PPy. The formation of SiO_2_ can be confirmed by the overlapping signals of Si and O. The overlap between C and Si signals is evidence that SiO_2_ is being spread equally throughout the carbon matrix. In addition, the overlapping of N, C, and Si elements with each other unequivocally demonstrates that PPy is dispersed consistently throughout the Nano-SiO_2_/C matrix. According to the findings, C, Si, O, and N elements are uniformly disseminated across the mixture. This confirms that the Nano-SiO_2_ and PPy nanoparticles are scattered throughout the carbon layer. From the SEM and EDS mapping observations of PPy@Nano-SiO_2_/C, it can be determined that the unique morphology of as-synthesized materials adheres to the proposed schematic diagram depicted in Figure 2i.

The N_2_ adsorption–desorption isotherms and associated pore-size distribution curves are shown in Figure S1a–c and Figure S1d–f, respectively, for Nano-SiO_2_, Nano-SiO_2_/C, and PPy@Nano-SiO_2_/C. The BET surface area of prepared materials is in the order of PPy@Nano-SiO_2_/C (117.15 m^2^/g) > Nano-SiO_2_ (68.24 m^2^/g) > Nano-SiO_2_/C (59.31 m^2^/g), whereas the corresponding pore volume is in the order of Nano-SiO_2_ (0.2914 cm^3^/g) > PPy@Nano-SiO_2_/C (0.1691 cm^3^/g) > Nano-SiO_2_/C (0.1177 cm^3^/g). Interestingly, similar type IV curves with hysteresis loops are observed for these three isotherms, as designated by the International Union of Pure and Applied Chemistry (IUPAC) [45]. Depending on the pore width, the type-IV isotherm contains a single hysteresis due to the absorption of mesopores, indicating that the pore width is wider than 4 nm [46]. In addition, a substantial hysteresis loop can indeed be noticed in the N_2_ adsorption–desorption isotherm, confirming that the produced materials have a high mesopore proportion. The pore size distribution was then described in terms of differential pore volume against differential log diameter (dV_p_/dlog(D)) in order to present pore size distributions, which are depicted in Figure S1d–f. The BJH theory was used to examine the pore size distribution in this study. All samples contained peaks with a range between 2 and 50 nm. The increase in dV_p_/dlog(D) peak intensity reflected a rise in porous density. After in situ polymerization of the pyrrole monomer, the number of pores in the PPy@Nano-SiO_2_/C sample emerged as having increased significantly. Therefore, the increase in surface area and pore density may have been caused by the porous nature of PPy-coated layers and particles. In particular, all synthesized samples exhibited a significant intensity peak between 1.5 and 15 nm. The analysis revealed that mesopores (2–50 nm) predominated in the three synthesized materials. Notably, the presence of mesopores in all materials enhances the electrolyte’s penetration, which ultimately decreases the lithium-ion diffusion path length, resulting in superior electrochemical performance at high current densities [47,48]. All data on the surface area and the porosity analysis of the synthesized materials are summarized in Table 2.

### 3.2. Electrochemical Performances

To assess the electrochemical performance, all prepared samples, Nano-SiO_2_, Nano-SiO_2_/C, and PPy@Nano-SiO_2_/C, were fabricated into working electrodes, for which SA brown algae was used as a binder, before being assembled into CR2016 half-coin battery cells with Li metal as a reference electrode. The fabricated cells were then aged for 12 h before being utilized for battery and electrochemical performance studies.

**Figure 3:**
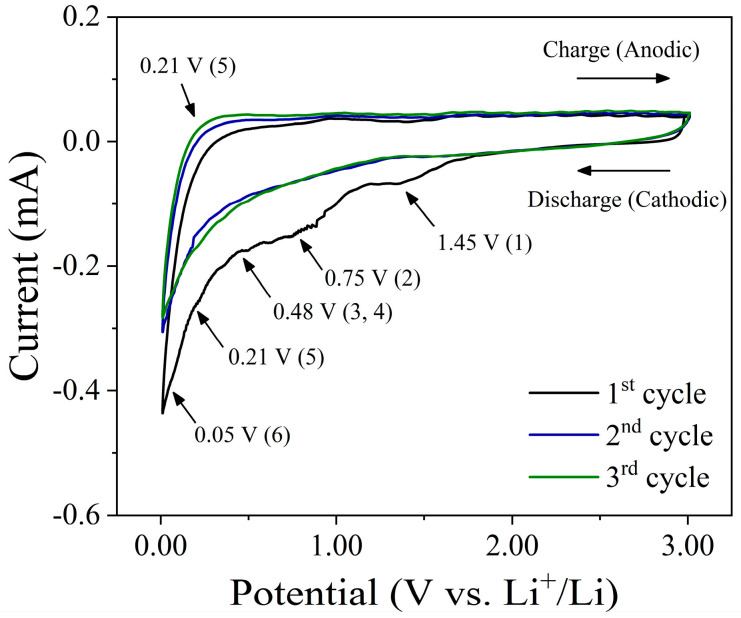
CV curves of prepared electrodes at the first three cycles of the PPy@Nano-SiO_2_/C electrode between 0.01 and 3.0 V at a scan rate of 0.1 mV s^−1^.

To evaluate the Li-storage mechanism, the cyclic voltammetry (CV) performance at a scan step of 0.1 mV s^−1^ over a circuit operating voltage of 0.01–3.00 V for the first three cycles was examined at the SA-PPy@Nano-SiO_2_/C electrode, as represented in Figure 3. There seem to be three detectable peaks (approximately 1.45 V, 0.75 V, and 0.48 V) within the first cathodic scans of CV plots, which are also represented in the initial discharge curve. The peak around 1.45 V is largely attributable to carbonate decomposition in the electrolyte, which is an unanticipated side reaction between the electrolyte and the electrode material [49]. In addition, the significant cathodic peak at 1.45 V, which reflects the irreversible reduction of SiO_2_ to Si, is represented in Equation (1), and the peak at 0.75 V can be referred to as the formation of the solid electrolyte interface (SEI) layer on the electrode surface as a consequence of the consumption of oxygenic functional groups, as shown in Equation (2) [50]. Upon the initial discharge of the fabricated electrode, amorphous SiO_2_ was transformed into Si, and Li_2_O was produced [10,51]. In addition, when the SiO_2_-based material was first discharged, Li_2_Si_2_O_5_ or Li_4_SiO_4_ would be created when the amorphous SiO_2_ turned into Si, as shown in Equations (3) and (4) [52,53]. The emergence of irreversible phases during the reaction required considerable capacity. However, the aforementioned effects diminish completely in subsequent cycles. Therefore, this reaction contributes to the electrode’s lithium storage capacity. In the second and third cycles, the CV curves become stable over the following scan cycles, reflecting the equivalent reversible behavior. As shown in Equation (5), the typical peaks associated with the reversible alloy/dealloy reactions of Si with Li^+^ involve cathodic peaks of approximately 0.21 V and anodic peaks of approximately 0.52 V, which correlate to the conversion between Si and Li*_x_*Si [12,54]. Multiple Li*_x_*Si phases coexisted during the lithiation (alloying) process, as shown by the appearance of a sharp reduction peak at 0.01–0.20 V in the cathodic scan [55]. Accordingly, this process increases the lithium storage capacity of the electrode. On the other hand, during the charge–discharge process, the peak at around ~0.5–0.01 V corresponds to the Li-ion intercalate and the extraction from graphite-like nanosized domains within the carbon sheets. Since the initial discharge process, lithium has intercalated first into defect sites (1.00 V) and then into nanographite-like domains (0.20 V) [56]. Thus, in subsequent cycles, the loss of capacity in the pseudo-graphitic nature of disordered hard carbons is mostly due to the decrease in lithium storage at defect sites, especially at carbon surfaces. The basic reaction mechanism of lithium-ion intercalation in the carbon material can be expressed in Equation (6). In the following scan, the second and third cycles almost overlap with each other, proving the high stability and reversibility of the lithiation and delithiation reactions. Obviously, the chemical reaction between PPy and Li was not observed, indicating that PPy may be an inactive material in this nanocomposite electrode. As aforementioned, all chemical reactions during charge–discharge processes could well be classified as follows:

Li^+^ + e^−^ + electrolyte → SEI(Li) (1)

SiO_2_ + 4Li^+^ + 4e^−^ → 2Li_2_O + Si (2)

5SiO_2_ + 4Li^+^ + 4e^−^ ↔ 2Li_2_Si_2_O_5_ + Si (3)

2SiO_2_ + 4Li^+^ + 4e^−^ → Li_4_SiO_4_ + Si (4)

Si + *x*Li^+^ + *x*e^−^ ↔ Li*_x_*Si (0 ≤ *x* ≤ 4.4) (5)

*x*Li^+^ + *x*e^−^ + C_6_ ↔ Li*_x_*C_6_ (6)

Figure S3 illustrates the galvanostatic charge–discharge curves for the PPy@Nano-SiO_2_/C electrode between the voltage cutoffs of 0.01 and 3.0 V at a current density of 0.3C (227.4 mA g^−1^). The initial discharge capacity of the fabricated electrodes was discovered to be 1342.5 mAh g^−1^. The first cycle’s charge capacity reduction is attributable to irreversible processes which is a side effect of electrolyte disintegration, the creation of a solid electrolyte interphase (SEI) layer, and unforeseen side reactions generated by the consumption of oxygenic functional groups corresponding to the CV result. The Coulombic efficiency (CE) of the second and third cycles improved to 86.0% and 97.7%, respectively, in subsequent cycles. These can be due to the decreasing effect of SEI film formation in the second cycle and their unique nanostructure’s ability to function as an effective anode, resulting in a nearly fully reversible Li^+^ reaction.

**Figure 4:**
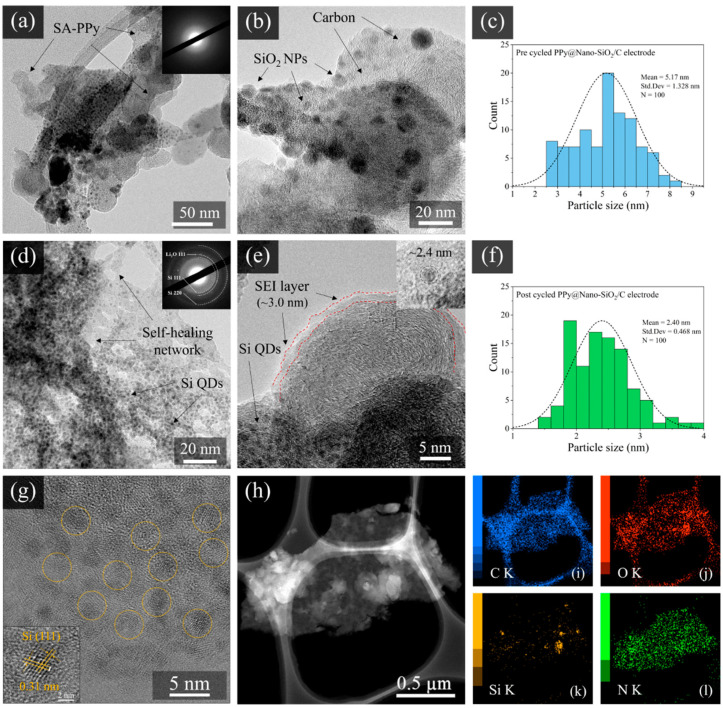
TEM images corresponding SAED patterns (inset) of pre-cycled (**a**,**b**) and post-cycled (**d**,**e**) PPy@Nano-SiO_2_/C electrodes and particle size histograms of SiO_2_ NPs (**c**) and Si QDs (**f**), HRTEM image of a single Si-QDs (**g**) showing the (111) lattice (inset), HAADF-STEM image (**h**), and EDS elemental mapping images of SA-PPy@Nano-SiO_2_/C: overlay of C K edge (**i**), O K edge (**j**), Si K edge (**k**), and N K edge (**l**).

To determine the morphology and structure of prepared PPy@Nano-SiO_2_/C nanocomposite electrodes before and after cycles, a TEM investigation was performed. Figure 4 displays TEM images of pre-cycled and post-cycled PPy@Nano-SiO_2_/C nanocomposite electrodes, along with selected area electron diffraction (SAED) analysis and particle diameter histograms of SiO_2_ nanoparticles (SiO_2_ NPs) and Si nanoparticles (Si NPs) in the carbon matrix and polymeric network. For the pre-cycled PPy@Nano-SiO_2_/C electrode, as depicted in Figure 4a,b, SiO_2_ NPs were principally observed in two configurations: agglomerated particles and dissociated SiO_2_ NPs. The particles are highly aggregated due to their small size and Van der Waal’s forces. However, the size of the cluster is still as large as the nanoscale. Furthermore, it is evident that the spherical SiO_2_ NPs developed predominantly within the carbon matrix. Figure 4c represents the corresponding particle size distribution of SiO_2_ NPs. It is evident that the diameter of the SiO_2_ NPs varied from 2 to 10 nm, with an average value of 5.17 nm. According to the SAED pattern (inset of Figure 4a), the amorphous structure was revealed. This can be identified by the amorphous nature of SiO_2_ and low-crystalline carbon. To examine the in-depth structure of carbon, the area of the carbon sheet was specifically taken, as shown in Figure S2. The TEM image (Figure S2a) revealed a thin sheet structure, with the SAED pattern corresponding to low-crystalline carbon. Thus, in the HRTEM observation, the lattice view of the carbon sheet is shown in Figure S2b, exposing the pseudo-graphitic nature of disordered hard carbon microstructures incorporating graphitic domains [57]. For the post-cycled PPy@Nano-SiO_2_/C electrode, Figure 4d demonstrates that the small particles were evenly distributed and embedded inside the carbon matrix and self-healing polymeric network. The SAED pattern depicted in the inset of Figure 4d exhibits three polycrystalline rings consistent with those of the Si (111), LiO_2_ (110), and Si (220) planes. These could verify the lithiation–delithiation mechanism that converts SiO_2_ NPs to Si quantum dots (Si QDs), whereas the SA-PPy polymer was transformed from spherical agglomerates to 3-dimensional networks. Figure 4e depicts the formation of the irreversible LiO_2_ reaction at this stage of the post-cycled electrode, which corresponds to the formation of the ~3 nm-thick SEI layer. A histogram analysis in Figure 4f reveals that the Si QDs are monodispersed and have an average size of 2.40 nm. Due to the ultrafine size of Si QDs, the high surface area interacting with Li-ion enables rapid lithiation and delithiation rates, enhancing specific capacity during long-term battery cycling. The crystallinity of the Si-NPs in the post-cycled electrode was further investigated by high-resolution transmission electron microscopy (HR-TEM image), as shown in Figure 4g. The HR-TEM displays Si-QDs distributed onto the carbon layer. We can observe the crystalline structure of QDs, which displays the (111) lattice sets with an interplanar spacing of ~3.10 Å, characteristic of Si. Figure 4h shows the HAADF-STEM image of a single sheet of SA-PPy@Nano-SiO_2_/C nanocomposite containing relatively heavy elements uniformly on the plate and lighter elements implied as the Si element of SiO_2_ nanoparticles. The result clearly elucidates the SA-PPy@Nano-SiO_2_/C nanocomposite structure, where the SiO_2_ nanoparticles are denser than the carbon layer and conductive polymer network. Figure 4i–l shows the results of the STEM-EDS elemental mapping analysis. The individual elemental maps show the relative positions of the synthesized materials, clearly identifying the plate structure visually decorated with carbon and SiO_2_, which correspond to the C K edge (Figure 4i), O K edge (Figure 4j), and Si K edge (Figure 4k), respectively. Moreover, this is unambiguous evidence of PPy network structure showing that N K edge (Figure 4l) signals are localized in a good dispersion on the SiO_2_/C nanocomposite area. From these advanced electron microscopy results, this work enriches the electrode engineering technology of nanocomposite materials using biomass waste recycling and opens up a new way to customize the self-healing anode for green and sustainable lithium-ion batteries.

**Figure 5:**
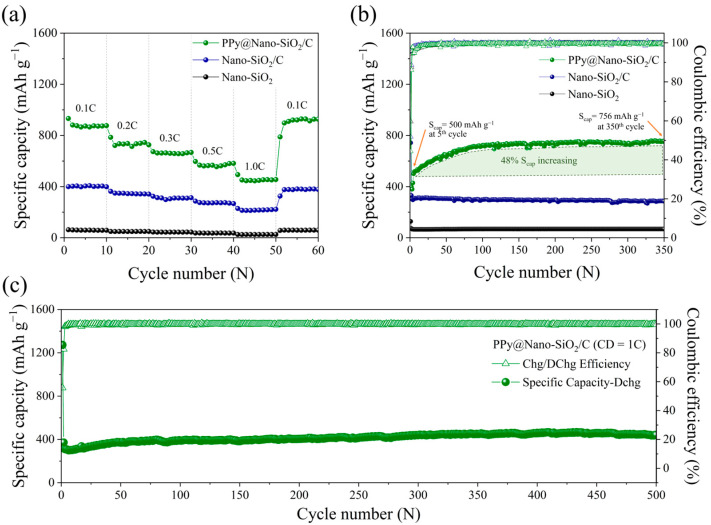
Battery performances of prepared electrodes: (**a**) rate cycle capability at different current densities in the range of 0.1C−1.0C, (**b**) comparative cycle performance and the corresponding Coulombic efficiency at a current density of 0.3C, (**c**) long-term cycle stability at a charging state of 1.0C for 500 cycles.

Figure 5a demonstrates the rate property on the lithiation (discharge) capacity of Nano-SiO_2_, Nano-SiO_2_/C, and PPy@Nano-SiO_2_/C at current densities ranging from 0.1 C to 1 C, which was carried out to investigate the rate capability. Even after rapid cycling between 0.1 C and 1.0 C, the PPy@Nano-SiO_2_/C electrodes offer the highest rate capability at 0.1 C. Evidently, PPy@Nano-SiO_2_/C has improved rate capability, as its specific capacity is greater than that of the fabricated electrodes at each rate. PPy@Nano-SiO_2_/C has reversible lithiation capacities of 879, 737, 663, 571, and 455 mAh g^−1^ at 0.1 C, 0.2 C, 0.3 C, 0.5 C, and 1.0 C, respectively. In fact, as the charge–discharge rates increased, the lithiation capabilities declined. After measuring the rate capability, the charge–discharge rate rapidly reverted to 0.1C, and the specific capacity can be recovered to 906 mAh g^−1^ with a 103% retention. This indicates that this higher percentage of retention may probably be due to the effect of SA-PPy’s self-healing character, which can improve not only the reversible capacity after many cycles but also the conductivity of composites during the charging–discharging process. The average specific capacity at various rates of current density and the percentage of retention at 0.1 C of prepared electrodes are recorded in Table S1. Owing to the fact that in rate evaluations, fabricated half-cells were only cycled for 10 cycles at each current rate, the performance difference between prepared electrodes was not significant. In order to determine the cycle duration of the three synthesized materials, extensive cycling tests were conducted. Figure 5b depicts the cycling stability profiles of prepared anodes measured over 350 cycles at a current density of 0.3 C. Importantly, considerable capacity fading typically emerges throughout the first cycle, most particularly due to SEI formation, the effect of irreversible capacity, and difficulty in extracting lithium from disordered materials during initial cycles, resulting in a loss of reversible capacity and a low CE. After 350 cycles, the specific capacities of Nano-SiO_2_, Nano-SiO_2_/C, and PPy@NanoSiO_2_/C electrodes reached 756, 285, and 68 mAh g^−1^, respectively, corresponding to CE values reinforced to nearly 100%. Surprisingly, the CE of all electrodes was immediate by the 5th cycle, and the overall cycling performance remained impressively consistent until the 350th cycle. In the scenario of PPy@Nano-SiO_2_/C electrodes, the reversible capacity improved from 500 mAh g^−1^ at the 5th cycle to 725 mAh g^−1^ at the 100th cycle. Following this, the specific capacity climbed marginally to 756 mAh g^−1^ compared to the fifth cycle, in which the specific capacity grew almost 48%. This remarkable occurrence may well be the result of three synergistic efforts. First, the reduction of SiO_2_ nanoparticles to ultra-fine SiQDs in the subsequent cycle and their well-distribution into the carbon and SA-PPy networks agreed with the TEM images in Figure 4d,g. This facilitated the electrochemical reaction with elevated and more stable active sites, resulting in a high specific capacity. Second, the design strategy of polymeric green nanocomposite manifests enhanced mechanical robustness that can inhibit SiQDs from being prematurely pulverized and electrical contract loss with that of the flexible backbone conductive network of dual SA-PPy polymer and restore the cracks from high-volume change of Si by filling as a result of its self-healing property. Together with the SA binder, the polymeric nanocomposite sustained the performance of PPy@Nano-SiO_2_/C anodes for over 350 cycles. Importantly, the dual protection of carbon and SA-PPy on SiO_2_ nanoparticles can significantly improve electrochemical performance, especially with respect to the excellent durability of PPy@Nano-SiO_2_/C electrodes. Third, the electrolyte may not completely wet the electrode prior to cycling. Once charging and discharging commence, the movement of lithium ions can allow the electrolyte to more thoroughly wet the PPy layer and SiO_2_/C. Consequently, in the initial few cycles, the capabilities rise with the number of cycles [58].

One of the most important determinants of whether applications of lithium batteries might well be realized is their long-stable life cycling with high-rate features. Consequently, it is essential to concentrate on the rapid charge–discharge performance of the electrode over its multiple cycles. At 1.0C charge–discharge rates, Figure 5c depicts the long-term cycling behavior of the PPy@Nano-SiO_2_/C electrode for 500 cycles. After five cycles, the PPy@Nano-SiO_2_/C anode exhibited an initial specific capacity of 306 mAh g^−1^; after 500 cycles, its specific capacity increased gradually to 441 mAh g^−1^. For the long-term test, this anode’s capacity retention at the 500th cycle was 46% higher than its initial capacity at the 5th cycle for the cycling assessment. These indicate that the PPy@Nano-SiO_2_/C has excellent cycling stability in order to use the anode because no capacity fading was observed after the 500th cycle. Consequently, the superior cycle performance of PPy@Nano-SiO_2_/C can be attributed to their SA-PPy network in the nanocomposite electrode together with a thin SEI layer, which has superior physical confinement on SiQDs, as evidenced in the microstructure of the cycled material in the TEM images in Figure 4d,e. As a result, the SA-PPy network offers enhanced electronic conductivity and ion transport efficiency. This study indicates that this fabricated SA-PPy@Nano-SiO_2_/C electrode has the ability to function well as an anode in sustainable LIBs for over 500 cycles.

**Figure 6:**
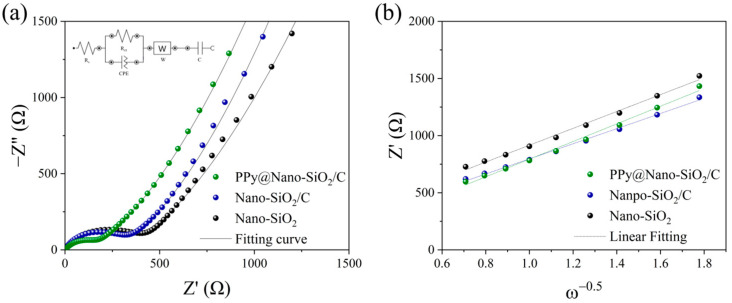
(**a**) Nyquist plots with a fitted equivalent circuit inset of prepared electrodes, and (**b**) Warburg coefficient plots for the initial state.

In order to comprehend the electrochemical kinetics of the fabricated electrodes, EIS measurements and equivalent impedance circuit fitting with the Nova 2.1 software have been conducted. The Nyquist plots of Nano-SiO_2_, Nano-SiO_2_/C, and PPy@Nano-SiO_2_/C pre-cycled electrodes are exhibited in Figure 6a, respectively. All measurements were performed on cells following a 12 h rest period. For all three electrodes, the plots can be considered to consist of a semicircle in the high-frequency region, followed by a sloping line in the middle to low-frequency region. The proposed circuit is an equivalent circuit (inset Figure 6a) to fit the Nyquist plots represented by solid lines to the experimental data. It is made up of an R symbolizing a resistor, which is comprised of interphase electronic contact resistance (R_s_) and charge transfer resistance (R_ct_) through the electrode–electrolyte interface, as well as a constant phase element (CPE) in parallel, a Warburg diffusion element (Z_w_), and a capacitor (C). Table 3 outlines the different prepared electrodes analyzed during the interpretation of Nyquist plots, for which the calculation method was followed in our previous report [49,59]. Considering the interfacial impedance of the pre-cycle electrode, the R_ct_ of the PPy@Nano-SiO_2_/C (156 Ω) shows the smallest resistance corresponding to the increment of lithium-ion diffusion, whereas the R_ct_ of the Nano-SiO_2_/C and Nano-SiO_2_ are 290 and 363 Ω, respectively, while the electrical conductivities (σ_ct_) of the electrode are in the order of PPy@Nano-SiO_2_/C > Nano-SiO_2_/C > Nano-SiO_2_. It means the PPy composite in materials has better effects on reducing interfacial impedance than carbon. Regarding the interfacial impedance of the pre-cycled electrode, the R_ct_ of the PPy@Nano-SiO_2_/C (156 Ω) exhibits the lowest resistance correlating to the increase in lithium-ion diffusion, whereas the R_ct_ of Nano-SiO_2_/C (290 Ω) and Nano-SiO_2_ (363 Ω) are, respectively, 290 and 363 Ω. This indicates that the PPy network composite is more effective than carbon at reducing interfacial impedance. The angular frequency (*ω*), which is related to *Z*′, can be used to compute the Warburg factor (σ*_w_*) throughout the low-frequency zone. Figure 6b depicts the plot of the line relationship between *Z*_re_ and *ω*^−1/2^. The Warburg factor (σ*_w_*) was calculated using the slope of the line of prepared electrodes. The lithium-ion diffusion coefficient $\left(D_{Li^{+}}\right)$, for which the proposed methodology and calculation were previously stated in our work [49], can therefore be computed from the Warburg region using Equation (7).

(7) $$D_{Li^{+}}=\frac{R^{2}T^{2}}{2A^{2}n^{4}F^{4}C^{2}\sigma_{W^{2}}}$$

where *R* is the universal gas constant, *T* is the absolute temperature, *F* is the Faraday constant, *n* is the number of electrons, *A* is the electrode area of the electrode, and *C* is the concentration of Li-ions in the electrolyte. According to Equation (8), σ*_w_* is the Warburg factor and *ω* is the angular frequency, which is related to *Z*′ and can be determined from the slope of the fitting line of *Z*′ vs. *ω*^−0.5^ in the EIS data at low frequencies, as shown in Figure 6b.

(8) $$Z_{re}=R_{e}+R_{ct}+\sigma_{w}\frac{1}{\sqrt{\omega }}$$

The Li^+^ diffusion coefficients ($D_{Li^{+}}$, cm^2^ s^−1^) of all cells are calculated using Equations (7) and (8), which $\sigma_{w}$ and $D_{Li^{+}}$ are shown in Table 3. The PPy@Nano-SiO_2_/C electrode has a higher $D_{Li^{+}}$ than that of the Nano-SiO_2_ and Nano-SiO_2_/C electrodes. It is evident that the $D_{Li^{+}}$ value is directly correlated to the rise in the components of carbon and PPy materials. This is due to the fact that SiO_2_, a major component, performs slower conversion reactions (Equations (2) and (4)) in the initial stage and subsequent alloying and dealloying reactions (Equation (5)) with Li ions than the PPy reaction and carbon intercalation (Equation (1)). The unique architecture of SA-PPy contributes to the high electrochemical performances, where 3D networks with good structural stability give sufficient space to achieve maximal contact across electrode and electrolyte, shorten the lithium-ion diffusion length, and alleviate volume change. Thus, the nitrogen lone-pair electron in the PPy backbone chain facilitating electron delocalization further enhances electrical conductivity, accelerates electron transport, and avoids SiQD agglomeration following reduction. As a result, these advantages contribute to an increase in $D_{Li^{+}}$ value, which is beneficial for improving battery performance, notably in circulation stability and specific capacity, by enabling significantly faster Li-ion transfer. As a consequence, these positive impacts on the success verify the excellent rate capability and cycle stability of PPy@Nano-SiO_2_/C electrode materials utilizing SA binder, which correlate to the previously discussed battery performances.

**Figure 7:**
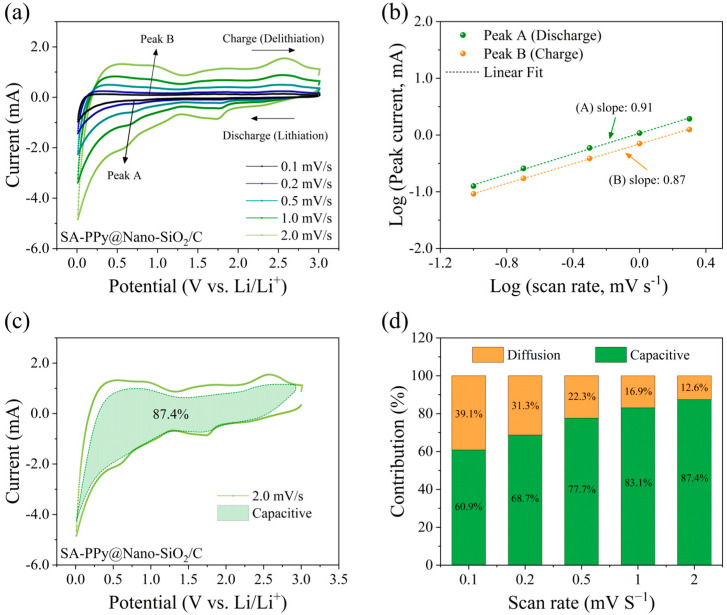
Dynamic analysis of PPy@Nano-SiO_2_/C electrode in a half-cell configuration: (**a**) CV curves recorded at different scan rates; (**b**) b-value of a relationship between the log (sweep rate, mV s^−1^) and log (peak current, mA) in the discharge and charge processes (marked as peak A and peak B in (**a**)), (**c**) CV curves at the scan rate of 2.0 mV s^−1^ with capacitive-controlled (green region) contribution; and (**d**) variation of capacitive and diffusion contribution at different scan rates.

To illustrate the detailed kinetic analysis and to evaluate the likely explanations for the improved rate capability, the as-synthesized SA-PPy@SiO_2_/C electrode was used as the anode for the suggested half-coin cells in order to separate the diffusion-controlled capacity and capacitive capacity. Figure 7a shows the CV curve of this electrode at various scan rates of 0.1 to 2.0 mV s^−1^ at room temperature, whose voltage window is 0.01–3.0 V. The capacitive and diffusive contributions can be used to evaluate the electrochemical kinetics of the electrode during high-rate performance. Comparable patterns have indeed been represented by the CV curves, and the peak intensities gradually rose as the scan rate increased. According to general knowledge, the measured current (*i*) and scan rate (*v*) satisfy the following power–law relationship in accordance with Equations [60,61]:

*i* = *av^b^* (9)

log(*i*) = *b*log(*v*) + log(*a*) (10)

where *i* represents the current’s magnitude, *v* indicates the scan rate, and *a* and *b* are variables. The capacity provided by the capacitive effect could be determined by constructing log(*i*)−log(*v*) curves according to Equation (10) and calculating the slope of the line (*b* value). According to prior research, as *b* approaches 0.5, a process is governed by complete diffusion, and when *b* approaches 1, the process is capacitive [62,63]. Therefore, by establishing the value of *b*, the battery’s major contribution could well be quantified. The value of *b* was 0.91 at peak A (discharge) and 0.87 at peak B (discharge). Figure 7b displays the linear fitting. This value indicated that the capacitive and diffusion-controlled mechanisms control the peak current in approximately equal measure. The overall capacitive contribution at a specified scan rate can be estimated by separating the capacitive and diffusion-controlled contributions at a defined voltage using the following Equations [24,64]:

*i* = *k*_1_*v* + *k*_2_*v*^1/2^ (11)

*i*/*v*^1/2^ = *k*_1_*v*^1/2^ + *k*_2_ (12)

where *k*_1_ and *k*_2_ are constants for a particular potential that could be obtained by linearly fitting *i*/*v*^1/2^ versus *v*^1/2^ under the defined potentials, and *v* is the scan rate for certain potentials. By identifying *k*_1_ as the slope and *k*_2_ as the intercept, capacitive and diffusion contributions could be determined. Integrating the green shaded area (*k*_1_*v*) with the measured currents (solid line) in Figure 7c demonstrates that capacitive interactions account for roughly 87.4% of the total current at 2.0 mV s^−1^ at the PPy@SiO_2_/C electrode. As a result, contribution ratios between the two methods were calculated at various scan rates. Figure 7d illustrates how the percentage of capacitive contribution grows with the scan rate. According to the current separation process, capacitive contributions rise significantly from 60.9% to 68.7%, 77.7%, 83.1%, and 87.4% of total capacitive contributions when the scan rate is increased by 0.1, 0.2, 0.5, 1.0, 1.5, and 2.0 mV s^−1^, respectively. This is not surprising since the role of capacitive contribution is growing, and pseudocapacitive contribution is important for ultrafine-reduced SiQDs and SA-PPy networks with a large surface area and a fast scan rate. These studies revealed that the capacitive-controlled process compensated for a large amount of the overall electrochemical reaction of the prepared electrode. As a consequence, the high capacitive contribution to the rate performance of the prepared electrode proved that the material has efficient redox reactions and activity that is independent of the electrochemical reaction rate [25]. Furthermore, this could also reflect that the conductive SA-PPy networks in the SA-PPy@SiO_2_/C electrode are a fundamental requirement and ensure a sustainable structure during charge–discharge processes [65,66].

**Figure 8:**
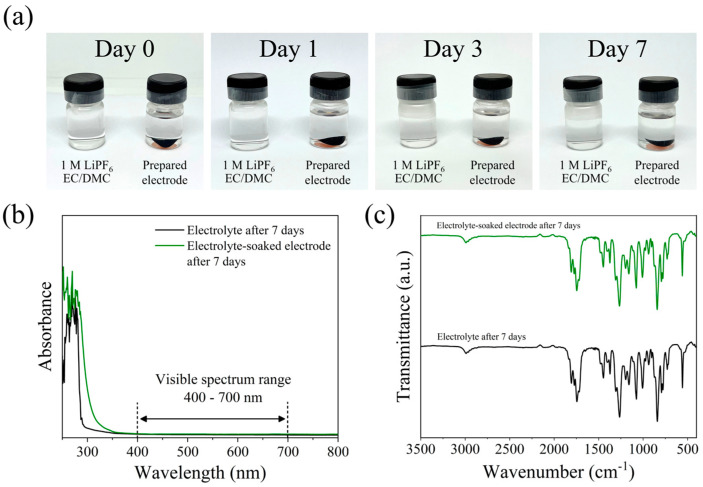
(**a**) Visualization dissolution tests of PPy@Nano-SiO_2_/C electrode in 1 M LiPF_6_ EC/DMC electrolyte, (**b**) Raman and (**c**) FTIR spectra of the collected electrolyte after being stored in the Ar-filled glovebox at 25 °C for 7 days.

To influence the effect of the SA-PPy framework on the Li-ion attraction of the PPy@Nano-SiO_2_/C electrode, the time-dependent transformation for 0, 1, 3, and 7 days during the dissolution experiments of the prepared anode in 1 M LiPF_6_ EC/DMC electrolyte was monitored, as demonstrated in Figure 8a. Determining their solubility by A snapshot revealed that even after seven days, the coloration of the solvent containing PPy@Nano-SiO_2_/C electrodes had not significantly altered. The electrolyte was still in a colorless and transparent solution and was the same shade as the electrolyte without an immersed electrode. Similarly, no noticeable evidence of electrode material detachment was discovered. As is evident from these, together with PPy, the high-binding SA binder efficiently prevented the surface delamination and dissolution of the prepared polymer-based materials. Figure 8b,c illustrate the UV-vis spectra and FTIR spectra, respectively, of the recovered electrolyte solution with and without the PPy@Nano-SiO_2_/C electrode to further clarify the suppression of the SA-PPy dissolution of active materials. After careful thought, these data revealed that the recorded spectra in each analysis were virtually identical. Accordingly, it can be concluded that SA-PPy@Nano-SiO_2_/C electrodes were insoluble in organic electrolytes. To verify the longevity of this electrode, the dissolution experiment in a 1 M LiPF_6_ electrolyte with/without the prepared electrode was conducted continuously for 60 days; however, the appearance remained equivalent to the initial day, and there was no evidence of electrode surface delamination, as shown in Figure S4.

**Figure 9:**
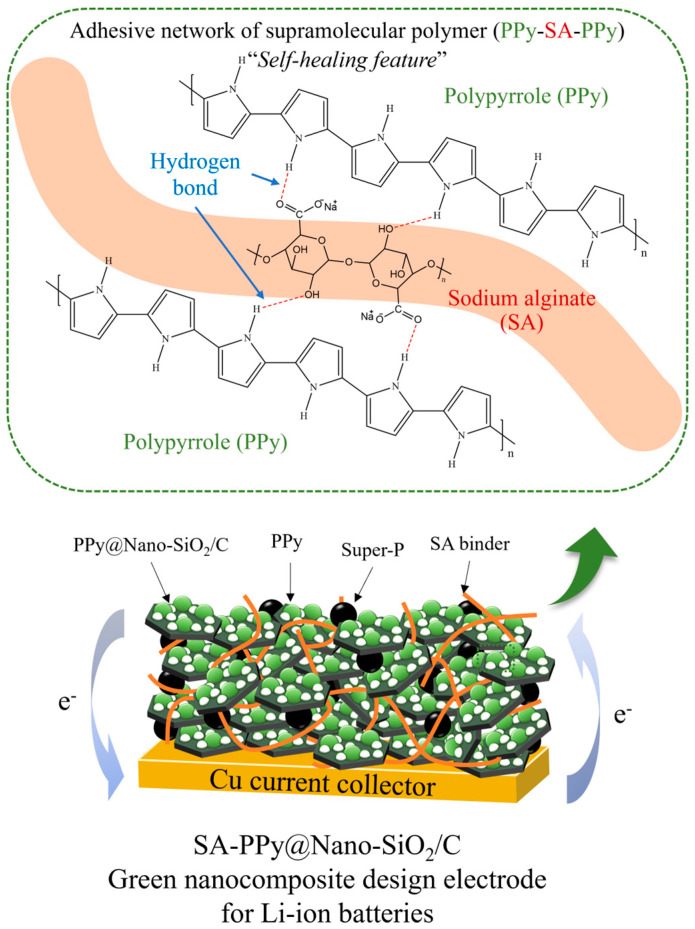
Schematic diagram of SA-PPy@Nano-SiO_2_/C electrode and adhesive polymer network of PPy-SA-PPy.

From all the discussions, this research has enhanced the efficiency of SA-PPy@Nano-SiO_2_/C anodes in LIBs by introducing the SiO_2_ particles with a self-healing composite polymer. The novel polymer blends of SA and PPy, which can serve as both conductive additives and binders, enhance stability and preserve a thin SEI layer. The SA-PPy system is constructed as an adhesive network of supramolecular polymers, each of which is connected by hydrogen bonds. Figure 9 depicts the schematic diagram of the SA-PPy@Nano-SiO_2_/C electrode and the adhesive polymer network of PPy-SA-PPy. As a result, the hybrid polymer structure maintains the electrical contract of SiO_2_/C particles and protects them from fracturing during multiple charge–discharge processes. Due to the hydrogen connections between the two polymers, the structure is capable of self-repair, as the polymers can gradually reconnect themselves if they disconnect at whatever juncture. In addition, the capability of PPy to significantly improve the anode’s conductivity and sustain a thin SEI, besides restricting the electrolytic degradation process of the electrolyte on the anode, could perhaps encourage enlarged lithium diffusion and minimize the interfacial impedance by limiting the excessive electrolyte decomposition on the anode’s surface. Notably, the hydrogen bond network generated between SA and PPy greatly improved the mechanical characteristics of the flexible composite polymer and offered robust cyclic stability to the SiO_2_-based anode. In comparison to the other electrochemical performances addressed, the PPy@Nano-SiO_2_/C green polymeric nanocomposites, in which SA was used as a binder, exhibit superior electrochemical performance. The comparison of SiO_2_-based nanocomposites with carbon and polymer materials is summarized in Table S2. In particular, the SiO_2_/C derived from SAL biomass is more cost-effective, simple, sustainable, and environmentally benign than competing SiO_2_/C sources. These are key advantages for industrial manufacturing. Therefore, the integration of PPy polymer and SA brown algae into green conductive polymeric nanocomposites can be greatly improved for multiple anode issues in LIBs. Finally, because of the effective functioning of their green composite design with unique structural morphological advantages and excellent electrochemical capabilities, this newly synthesized PPy@Nano-SiO_2_/C material and its green nanocomposite design pave the way for the next generation of sustainable energy storage systems and can be considered a promising anode material for advanced applications.

## 4. Conclusions

This research achieved success in developing an ecologically friendly and sustainable PPy@SiO_2_/C nanocomposite anode material for LIBs. *Streblus asper* leaves, a biomass waste, were used not only to produce carbon-based materials possessing conductive properties, but also to contain self-assembled SiO_2_ nanoparticles in the form of SiO_2_/C that functioned as a greater specific capacity in anodes. Then, they were introduced into a conductive polymer network employing PPy in combination with SA binder to function as mechanically robust and self-healing nanocomposites in SA-PPy@SiO_2_/C. In terms of electrochemical performance, the SA-PPy@SiO_2_/C demonstrates outstanding achievement, particularly in terms of specific capacity and cycle stability. At a current density of 0.3C after 350 cycles, the specific capacity remains high, roughly 756 mAh g^−1^, with a Coulomb efficiency of up to 99.5%. This nanocomposite electrode has acceptable rate performance and cycle stability for a long-term cycle of over 500 cycles, while this material has better impacts on interfacial impedance and Li diffusion than those electrodes. The excellent electrochemical properties can be attributed to the unique properties and self-healing features of the SA-PPy 3D network assembly, which improve the conductivity of composites during the charging–discharging process. Besides that, the carbon layer, in conjunction with the flexible SA-PPy can be thought of as a cushion to prevent the expansion of SiO_2_ microspheres. When compared to traditional anodes, the SA-PPy@SiO_2_/C with a green polymer nanocomposite design has superior characteristics, is more environmentally benign, and is more sustainable because it can potentially be mass-produced with plenty of resources in a low-cost manner for the next generation of LIBs and energy storage fields.

## Figures and Tables

**Table 1:** The calculated weight percentage of as-prepared materials from TGA and their theoretical specific capacity calculation.

Materials	Theoretical Specific Capacity (mAh g^−1^)	The Calculated Weight Percentage of As-Prepared Materials (wt.%)		
		Nano-SiO_2_	Nano-SiO_2_/C	PPy@Nano-SiO_2_/C
SiO_2_	1965	100.0%	38.1%	23.3%
Carbon	372	-	61.9%	37.8%
Polypyrrole	412	-	-	38.9%
		Calculated theoretical specific capacity (mAh g^−1^)		
		1965	979	758

**Table 2:** The results of the surface area and the porosity analysis of the synthesized materials.

Synthesized Materials	Specific Surface Area, S (m^2^/g)	BET C-Constant	Pore Volume, V_p_, (cm^3^/g)	Average Pore Radius (Å)	Average Pore Diameter (nm)
Nano-SiO_2_	68.24	23.26	0.2914	85.42	17.084
Nano-SiO_2_/C	59.31	33.38	0.1177	39.67	7.934
PPy@Nano-SiO_2_/C	117.15	4.41	0.1691	28.87	5.774

**Table 3:** Fitted values of the corresponding parameters of the prepared anode electrodes.

Prepared Electrodes	R_s_ (Ω)	R_ct_ (Ω)	σ_ct_ (S/cm)	σ_W_ (Ω/s^1/2^)	D_Li_^+^ (cm^2^/s^1/2^)
Nano-SiO_2_	0.382	363	2.75 × 10^−7^	737.68	2.36 × 10^−19^
Nano-SiO_2_/C	0.207	290	3.45 × 10^−7^	661.60	1.12 × 10^−17^
PPy@Nano-SiO_2_/C	0.107	156	6.41 × 10^−7^	771.94	4.65 × 10^−17^

## Data Availability

Data will be made available on request. The data are not publicly available due to ongoing study and commercial restrictions.

## Supplementary Materials

The following supporting information can be downloaded at: https://www.mdpi.com/article/10.3390/polym16101414/s1, Table S1: Average specific capacity at various rates of current density and the percentage of retention at 0.1C of prepared electrodes; Table S2: Comparison of the specific capacity and cycle performance of SiO_2_-based composites with carbon and polymer materials as anode materials in LIBs Materials; Figure S1: BET analysis of the nitrogen adsorption–desorption isotherm corresponding pore-size distribution curves inset: (Figure S1a,d) Nano-SiO_2_, (Figure S1b,e) Nano-SiO_2_/C, and (Figure S1c,f) PPy@Nano-SiO_2_/C; Figure S2: (a) TEM image with SAED pattern inset and (b) HRTEM image with lattice view at carbon sheet of Nano-SiO_2_/C nanocomposite; Figure S3: The GCD profiles at the first three cycles of the SA-PPy@Nano-SiO_2_/C electrode; Figure S4: The longevity verification experiment in a 1 M LiPF_6_ electrolyte with/without the prepared electrode after 60 days.
