# Supplementary material for: Biomass Waste Utilization as Nanocomposite Anodes through Conductive Polymers Strengthened SiO2/C from Streblus asper Leaves for Sustainable Energy Storages
Source: Polymers (Basel). 2024 May 16;16(10):1414. doi: 10.3390/polym16101414 (PMC11125036; doi:10.3390/polym16101414)
Supplement: Supplementary file 1 [file polymers-16-01414-s001.zip › polymers-2968736-supplementary.pdf]

Supporting Information

Biomass Waste Utilization as Nanocomposite Anodes through Conductive Polymers Strengthened SiO_2/C from *Streblus asper* Leaves for Sustainable Energy Storages

Thanapat Autthawong [1,2,3], Natthakan Ratsameetammajak [2,4], Kittiched Khunpakdee [2,4], Mitsutaka Haruta [5], Torranin Chairuangsri [6] and Thapanee Sarakonsri [2,3,4,*]

Table S1. Average specific capacity at various rates of current density and the percentage of retention at 0.1C of prepared electrodes.

Current density (C-rate)	Average specific capacity (mAh g^{-1})		
	Nano-SiO_2	Nano-SiO_2/C	PPy@Nano-SiO_2/C
0.1C	60	403	879
0.2C	49	347	737
0.3C	44	311	663
0.5C	37	274	571
1.0C	26	218	455
0.1C	59	381	906
% Capacity retention at 0.1C	98%	95%	103%

Table S2. Comparison of the specific capacity and cycle performance of SiO_2-based composites with carbon and polymer materials as anode materials in LIBs Materials

Anode materials in LIBs	Year	Silica source	Silica content (wt.%)	Battery cycling performance			Ref.
				Current density (mA g^{-1})	Specific capacity (mAh g^{-1})	Number cycle (N)	
SiO_2-based composites with carbon							
3D interconnected network SiO_2–C/NCs	2016	Bamboo leaf	~51	200	294	180	[68]
SiO_2@graphite composites	2017	Sewage Sludge	-	100<br>1000	433<br>244	100<br>500	[69]
SiO_2 nanoparticles embedded carbon matrix	2017	APTES	~40%	100<br>200	888<br>547	100<br>10	[70]
SiO_2/C/graphene spheres	2017	TEOS	43%	50	605	100	[71]
Yolk structure porous C/SiO_2/C	2018	TEOS	~68%	50<br>300	1027<br>400	80<br>10	[72]
Carbon coated SiO_2	2019	Diatomic frustules	~54%	200	600	50	[73]
Yolk-shell silica@carbon frameworks	2019	TEOS	~57%	100<br>500	630<br>373	150<br>1000	[74]
SiO_2 nanoparticles/ carbon plate	2020	Rice husk	-	100	176	100	[75]
C/SiO_2 composites	2020	Rice husk	~38%	100	420	100	[76]
Carbon coated ball milled SiO_2	2020	Diatomaceous earth	~87%	100	840	100	[77]
Hierarchically porous SiO_2/N-doped carbon	2020	Rice husk	-	100	780	100	[78]
SiO_2@graphene-like carbon nanocomposites	2021	Corn cob core	~19%	200	125	200	[79]
SiO_2 nanotubes coated N-doped carbon layers	2021	TEOS	~65%	100<br>200	781<br>680	200<br>10	[80]
Carbon-coated SiO_2/C composites	2022	Rice husk	~55%	100	520	100	[81]
SiO_2/N-doped graphene nanocomposites	2022	Rice husk	~10%	288 (C/3)	385	150	[82]
SiO_2-based composites with polymer							
Hollow triple shelled SiO_2/TiO_2/PPy nanospheres	2014	TEOS	~2%	44	433	50	[83]
PPy coated on SiO_2 encapsulated porous carbon nanofibers	2021	TEOS	~20%	500	300	300	[84]
SiO_2@cPANI/cTOCNFs	2022	Nanosilica	~59%	100<br>1000	1103<br>302	200<br>1000	[85]
PANI-coated nano-silica@rGO	2022	Rice husk	~6%	400	680	500	[86]
Polypyrrole/SnO_2@SiO_2	2022	TEOS	-	0.1C	676	100	[41]
SiO_2-rGO@PPy	2022	Rice husk	~17%	100	523	250	[65]
PPy@Nano-SiO_2/C	2023	*Streblus asper* leaf	~23%	76 (0.1C)<br>228 (0.3C)<br>758 (1.0C)	927<br>756<br>441	10<br>350<br>500	This work

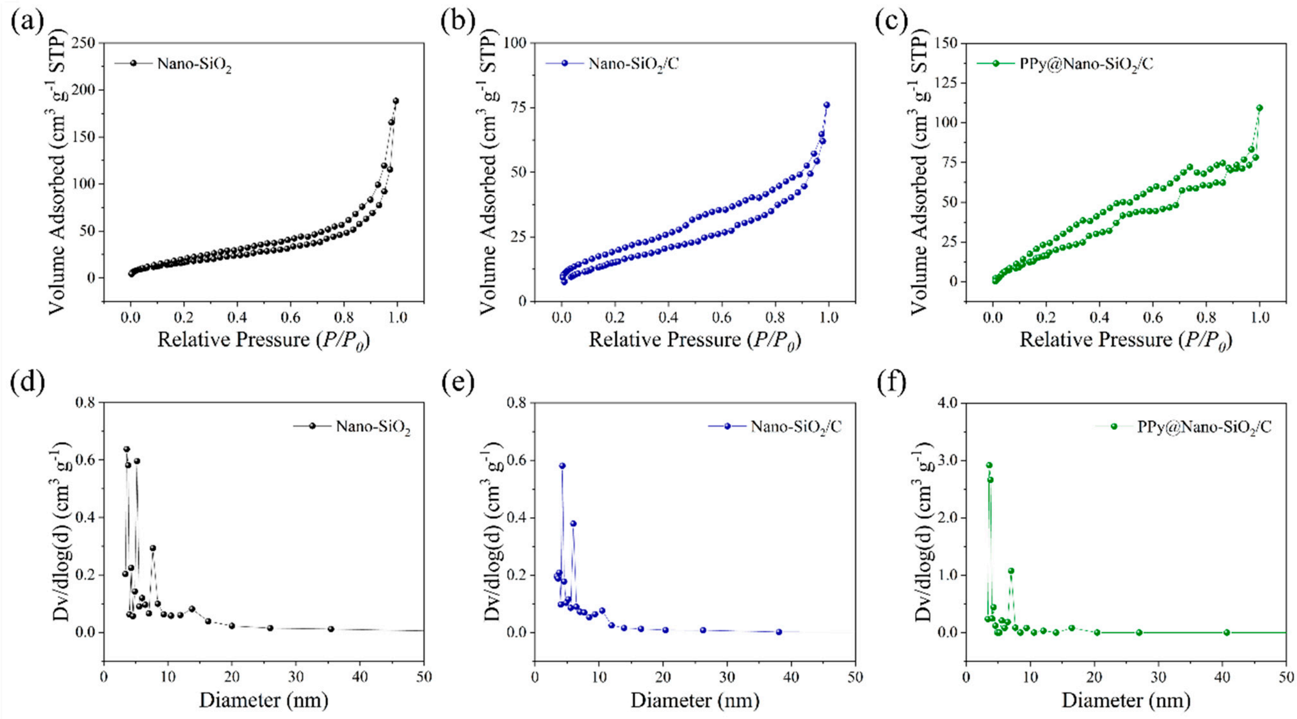

Figure S1. BET analysis of the nitrogen adsorption-desorption isotherm corresponding pore-size distribution curves inset: (a,d) Nano-SiO_2, (b,e) Nano-SiO_2/C, and (c,f) PPy@Nano-SiO_2/C.

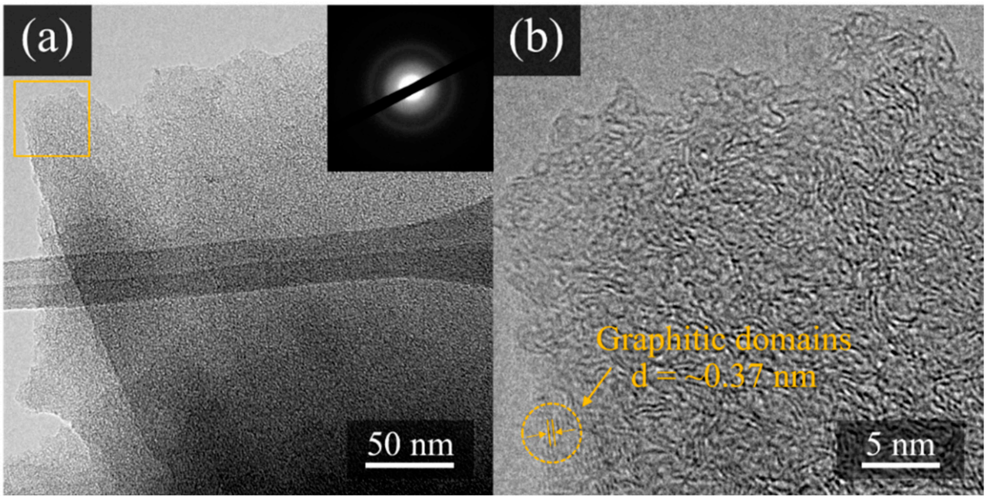

Figure S2. (a) TEM image with SAED pattern inset and (b) HRTEM image with lattice view at carbon sheet of Nano-SiO_2/C nanocomposite

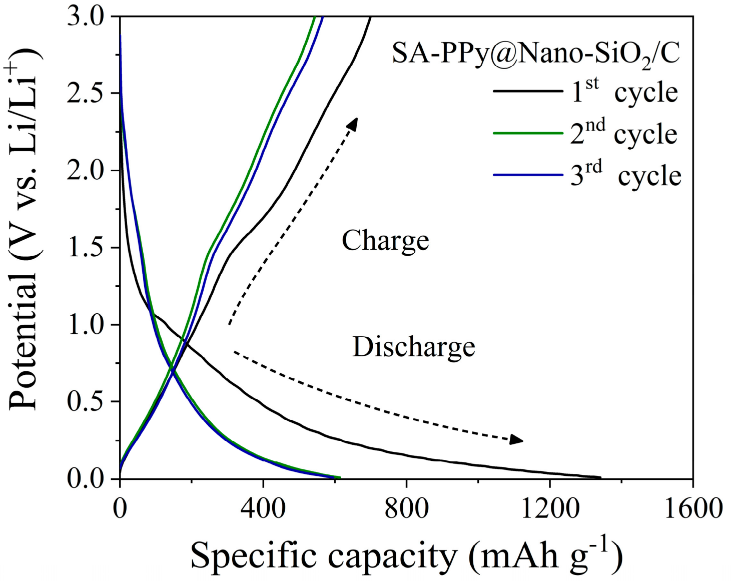

Figure S3. the galvanostatic charge–discharge (GCD) profiles at the first three cycles of the fabricated SA-PPy@Nano-SiO_2/C electrode

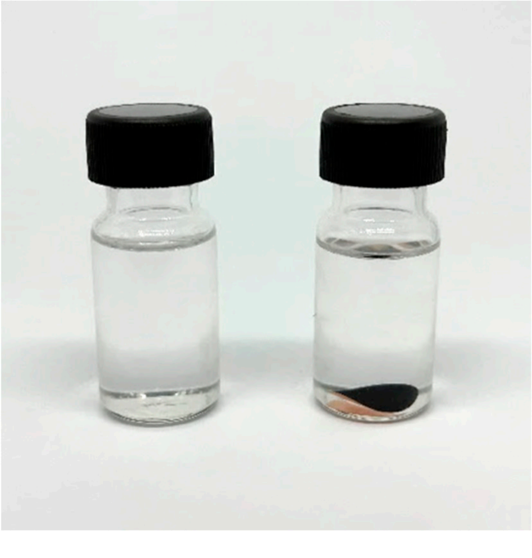

Figure S4. The longevity verification experiment in a 1 M $LiPF_6$ electrolyte with/without the prepared electrode after 60 days
